# Comparative Effects of Applied Behavior Analysis on Male and Female Individuals With Autism Spectrum Disorder

**DOI:** 10.7759/cureus.59802

**Published:** 2024-05-07

**Authors:** Tami Peterson, Jessica Dodson, Robert Sherwin, Frederick Strale,

**Affiliations:** 1 Hyperbaric Oxygen Therapy, The Oxford Center, Brighton, USA; 2 Applied Behavior Analysis, The Oxford Center, Brighton, USA; 3 Hyperbaric Oxygen Therapy, Wayne State University School of Medicine, Detroit, USA; 4 Biostatistics, The Oxford Center, Brighton, USA

**Keywords:** gender differences, treatment effects, cumulative target behaviors, applied behavior analysis, autism spectrum disorder (asd)

## Abstract

Introduction

Current evidence-based treatments for autism spectrum disorder (ASD) are based on applied behavior analysis (ABA). However, research on gender differences in ABA therapy response is limited. This study seeks to (1) confirm the 4:1 male-to-female ratio reported in the literature and (2) identify any possible gender differences in target behaviors over seven timepoints measured every two weeks.

Materials and methods

For three months, from March 19, 2023, to June 11, 2023, a team of 3-5 behavioral technicians per individual collected daily data on general target mastery for 100 individuals with ASD treated with ABA. Data was collected at seven timepoints every two weeks. Descriptive demographics were computed. Two independent sample t-tests were performed to determine significant or nonsignificant gender differences with the seven timepoint variables.

Results

Nonstatistically significant gender differences (p > .05) were found on all seven cumulative target behavior timepoints measured at two-week intervals. For targets mastered Time 1, baseline between males and females, there was no significant difference in the means for males (M = 1.0571, SD = 1.9196) and females (M = 2.0455, SD = 3.9457) (t(90) = -1.591, p = 0.115, confidence interval (CI) = -2.2223, 0.2456, d = -0.389). For targets mastered Time 2, two weeks between males and females, there was no significant difference in the means for males (M = 3.7132; SD = 4.5065) and females (M = 4.0682, SD = 5.1508) (t(88) = -0.310, p = 0.757, CI = -2.6305, 1.92056, d = -0.076). For targets mastered Time 3, four weeks between males and females, there was no significant difference in the means for males (M = 7.0956; SD = 8.7781) and females (M = 8.6136; SD = 11.2799) (t(88) = -0.656, p = 0.514, CI = -6.1173, 3.0811, d = -0.161). For targets mastered Time 4, six weeks between males and females, there was no significant difference in the means for males (M = 13.1728, SD = 16.2003) and females (M = 13.0682, SD = 16.9272) (t(88) = 0.026, p = 0.979, CI = -7.8779, 8.0871, d = 0.006). For targets mastered Time 5, eight weeks between males and females, there was no significant difference in the means for males (M = 17.2096; SD = 18.8546) and females (M = 17.4286, SD = 22.1683) (t(87) = -0.045, p = 0.965, CI = -9.9773, 9.5393, d = -0.011). For targets mastered Time 6, 10 weeks between males and females, there was no significant difference in the means for males (M = 21.0074, SD = 21.3329) and females (M = 20.6818, SD = 26.1231) (t(88) = 0.059, p = 0.953, CI = -10.6752, 11.3262, d = 0.014). For targets mastered Time 7, 12 weeks between males and females, there was no significant difference in the means for males (M = 26.1196, SD = 24.2235) and females (M = 29.6364, SD = 33.7406) (t(89) = -0.536, p = 0.593, CI = -16.5431, 9.5094, d = -0.131).

Conclusions

The study indicates that ABA treatments may be equally beneficial for both genders with ASD, showing no significant gender differences. However, the broad CIs in this study imply a level of statistical uncertainty, indicating potential gender differences, suggesting the results may not be uniform across genders. These findings challenge assumptions on gender-specific treatment responses, implying that ABA treatments shouldn’t be recommended based on gender. Instead, individual needs should guide treatment recommendations. Future research could consider other response moderators like age, ASD severity, or coexisting mental health conditions.

## Introduction

According to the Autism and Developmental Disabilities Monitoring (ADDM) Network of the Centers for Disease Control and Prevention (CDC), it is estimated that one out of every 36 children is diagnosed with autism spectrum disorder (ASD). ASD is observed across all ethnic, racial, and socioeconomic groups. Interestingly, the prevalence of ASD is almost four times higher in boys (4:1) compared to girls [1]. 

Peterson et al. found that ABA therapy significantly improved patient behavior over time and noted that applied behavior analysis (ABA) therapy is widely acknowledged as the benchmark for treating ASD [2]. This recognition stems from years of research and a substantial body of corroborating evidence [2]. Yu et al. conducted a meta-analysis of 14 randomized control trials involving 555 participants, revealing that ABA had a moderate to high impact, offering significant advantages for children with ASD [3]. 

Makrygianni et al. analyzed 29 studies and concluded that ABA programs were moderate to highly effective, providing substantial benefits to children with ASD [4]. In a randomized controlled trial by Dixon et al. involving 28 children with autism, it was reported that the most significant changes in intelligence scores were observed in participants in the comprehensive ABA group [5]. 

Rodgers et al. assessed 25 studies in a systematic review and meta-analysis of 20 studies to determine the clinical effectiveness of early intensive ABA-based interventions for children with autism [6]. They observed considerable heterogeneity, with effects varying significantly across studies. They noted that due to limited data, the impact of the intervention on autism symptom severity, language development, and school placement remains uncertain. The long-term effects are also unclear due to a lack of follow-up data [6]. 

Eckes and colleagues assessed the effects of ABA on developmental outcomes in children with ASD and the associated parental stress [7]. This assessment was based on a review of 11 studies involving 632 participants. Compared to standard treatment or minimal to no treatment, comprehensive ABA-based interventions demonstrated medium effects on intellectual functioning and adaptive behavior. However, there were no improvements beyond the control groups in language abilities, symptom severity, or parental stress [7]. 

Gitimoghaddam et al. systematically searched seven online databases to identify peer-reviewed English-language studies examining the impact of ABA on health outcomes [8]. They categorized the measured ABA outcomes into eight categories: cognitive, language, social/communication, problem behavior, adaptive behavior, emotional, autism symptoms, and quality of life [8]. 

The results of the study were categorized based on gender. In the studies that focused solely on females, problem behavior was the most frequently examined outcome, accounting for 33% of the cases. This was followed by social/communication outcomes, which made up 23% of the cases. These outcomes showed improvements at 85% and 67% of the time, respectively [8].

In studies that only involved males, the most frequently examined outcome was language, which accounted for 26% of the cases. This was followed by cognitive and social/communication outcomes, each making up 21% of the cases. These outcomes showed improvements at 62%, 66%, and 59% of the time, respectively [8].

In studies that included both genders, the most frequently examined outcomes were language (25%), cognitive (22%), and social/communication (21%). These outcomes showed improvements at 65%, 71%, and 67% of the time, respectively [8].

Anderson and Carr underscored numerous meta-analyses, systematic reviews, and cost-benefit analyses that attested to the effectiveness of ABA-based interventions for individuals with autism [9]. However, they noted an “efficacy-effectiveness gap” due to individual heterogeneity, neurodiversity, reduced compliance levels, general medical presentation rather than specialist settings, less standardized and monitored treatments, and cost pressures [9].

Mathur and Renz focused on the importance and implications of future research affirming neurodiversity with qualitative studies with interview of patient experiences and how ABA procedures can be improved and large and small quantitative studies across a diverse array of regions and cultures, with emphasis on personal identity and happiness [10].

Stalford et al. emphasized the importance of positive behavioral support (PBS) training grounded in ABA principles, despite anti-ABA anecdotal bias fueled by misinterpretation and unsubstantiated anecdotal claims [11]. 

Despite the strong evidence supporting its effectiveness, the adoption of evidence-based procedures remains low. Misunderstandings and misconceptions about ABA are widespread, and challenges in determining appropriate research methods to evaluate the effectiveness of individualized interventions contribute to disagreements about what constitutes evidence [12]. ABA has been widely recognized as the gold standard for treating ASD, a recognition that is backed by decades of research and a substantial body of supporting evidence. ABA is a popular and widely preferred method of treatment. The ranking or placement of therapies for ABA can vary based on several factors, such as the child's individual needs. Other treatments include speech, physical, occupational, nutritional, and cognitive behavioral therapy, play therapy, social skills training, and developmental approaches [12,13]. 

Gender differences with ABA efficacy research

Results on gender differences relative to the impacts of ABA on target behaviors with autistic individuals are limited. ASD presents differently, including variations in symptoms, severity, and co-occurring conditions. This heterogeneity can influence how an individual responds to ABA therapy [9]. Considerations of culture and neurodiversity are essential and can significantly influence research, practice, and discussions among stakeholder groups [10]. The wide range of procedures in ABA makes analyzing the parameters and components that contribute to its effectiveness quite complex [11-13]. 

Cariveau et al. reported on gender differences in core symptoms, associated features, and treatment response in a convenience sample of 682 youth (585 males, 97 females) with ASD. The sample included participants (mean = 7.4 years; range 3-17 years) from six federally funded, multisite, randomized clinical trials. They found that youth with ASD's clinical characteristics and response to treatment showed no significant gender differences [14]. 

Tiura et al. found that gender predicted behavioral growth rates due to ABA interventions. Male subjects tended to improve more quickly in adaptive behavior and physical development [15]. Although this finding was significant, there was a great discrepancy in the number of male (n = 27) and female (n = 8) participants (3.4:1) in this study. This study's small sample of female participants may have resulted in limited variability. The researchers also report that other studies have not found gender predictive of treatment outcomes [15-17]. 

Khasawneh had 100 autistic children in their experimental study [18]. Their experimental cohort had 50 young people, with a mean age of 6.8 years (standard deviation = 1.2), with 30 males and 20 females. Like the experimental group, the control group included 50 young people with a somewhat younger mean age of 6.5 years (SD = 1.5). There were 35 males and 15 females in this control grouping. They discovered that males had significantly lower stereotyped behavior scale (SBS) scores than females (p = 0.039). This finding indicated that the use of evidence-based ABA treatments was more successful in reducing stereotypical behaviors among male participants than female participants [18]. 

Study objectives 

Given the limited research specifically examining gender disparities in response to ABA therapy in individuals with ASD, the objectives of this study aim to (1) produce confirmatory evidence for the 4:1 male-to-female ratio reported in the scientific literature and to (2) find evidence that may indicate significant or nonsignificant gender difference within this sample of individuals with ASD. 

## Materials and methods

Participants 

A team of three to five behavioral technicians per child gathered general target mastery data daily for 100 individuals with autism who received 12 weeks of ABA interventions. This data was collected using a large N design through retrospective chart reviews within the “Catalyst” tracking software [19]. From March 19, 2023, to June 11, 2023, behavior analysts implemented a mixed model of discrete trial training, mass trials, and naturalistic environment treatment for three months. General target mastery data was compiled for 89 children and four adults, with seven instances of missing data. 

Inclusion/exclusion criteria 

This study included males and females diagnosed with ASD by a psychiatrist, psychologist, or primary care physician between the ages of one and 73 years and medically approved for treatment. Excluded from this study were individuals without an ASD diagnosis, those with a medical condition or disability that could make ABA therapy unsafe, those with a history of abuse, neglect, or trauma that could hinder their ability to benefit from ABA therapy, those who were undergoing another intervention incompatible with ABA therapy, and cases where the family and the provider were unable to resolve significant issues related to the treatment plan. 

Data acquisition 

Catalyst, a commercial electronic data collection tool, aids interventionists in capturing and analyzing vast amounts of behavioral data, mirroring the traditional paper data collection methods used by behavior technicians. Board Certified Behavior Analysts (BCBAs) devised a treatment plan for each individual, implementing programs and data collection methodologies for behavior reduction and skill acquisition [19,20]. 

Behavior technicians assigned to specific individuals with autism utilized real-time data-stamping procedures to record data the moment the behavior was observed. Using a portable electronic device (an iPad; Cupertino, CA: Apple Inc.), the behavior technician formulated an operational definition for the problematic behavior and chose continuous (frequency, duration) measurement systems. This data was then accessible online for researchers to analyze and report. 

All individuals with autism were treated at The Oxford Centers (TOCs) in Brighton and Troy, MI. TOCs specialize in the mixed methods approach to ABA, utilizing discrete trial training, mass trials, and naturalistic environment training treatment modalities. Before training, each subject received a treatment plan developed by one of eight BCBAs, tailored to the individual’s needs and goals. 

The individual was assigned to one of 83 behavioral technicians and had a team of three to five behavioral technicians over the three months. Suitable materials were chosen and arranged in rooms where individual discrete trial training and mass trials occurred or in a naturalistic setting where the participant interacted with others and experienced functional and meaningful real-world situations. Each behavioral technician was assigned to a different participant daily, providing, on average, four to seven hours of treatment per day for a minimum of 25 hours a week. 

Behavioral technician teams collected specific behavioral and skill data related to antecedents, behavior, and consequences of behavior. They monitored progress, noted the fading of prompts and reinforcements as the participant attempted to master the skill, and assessed whether the participant was generalizing and maintaining it. Data was entered into a handheld “Catalyst” database and aggregated and updated daily into a central database. 

Dependent measures 

The dependent variable in this study was the composite scores from multiple raters for the number of mastered general target behaviors. These scores were measured at seven different timepoints every two weeks for three months. These “general aggregate target behaviors,” as defined by BCBAs and behavioral technicians at The Oxford Center, encompassed daily living skills. These skills included daily routines, organization, time management, eating-related skills, toileting, and hygiene routines. 

Participants were taught expressive communication skills, which involved speaking with words and phrases, expanding vocalizations to use more complex vocabulary, improving conversational skills, greeting people, responding to greetings, asking for assistance, and making requests. Emphasis was also placed on receptive language skills, such as following directions and identifying stimuli upon request. 

Training was provided for social skills, including taking turns playing with friends, sharing, displaying assertiveness, interacting with peers, and responding appropriately to new people. Community skills were practiced in naturalistic environments, such as responding to a cashier in a store, purchasing items, managing money, shopping for groceries, ordering food in a restaurant, speaking to a police officer, walking safely on a sidewalk, playing safely at a park, and safety skills with strangers. 

The independent variable was time, with seven levels (Time 1, baseline; Time 2, after two weeks; Time 3, after four weeks; Time 4, after six weeks; Time 5, after eight weeks; Time 6, after 10 weeks; and Time 7, after 12 weeks). Given the variation in each participant’s treatment plan, the administered treatment generally consisted of a mixed model of discrete trial training combined with massed trial instruction and naturalistic environment treatment. Reinforcers were chosen for their strength, clear contingencies, and repetition to teach new behaviors. 

Luiselli noted that naturalistic teaching promotes the generalization of skills to everyday settings where those skills are required, thus enhancing the generalization of language, social, and play skills [21]. Compared with more structured approaches, naturalistic teaching better generalizes critical skills to the natural setting. These procedures occur within the context of everyday activities, making learning more enjoyable and enhancing the individual’s willingness to engage in learning. 

This instills confidence that these procedures are a viable, evidence-based method in providing therapy to individuals with autism. ABA interventionists teach responses, creating contact with natural reinforcers, allowing the individual’s interests to direct and pace teaching. Naturalistic environments also embed education within everyday activities, incorporating prompts to be transported to new situations. Some skills can be learned in a controlled setting before transitioning to a naturalistic setting [21]. 

The dataset used for this study assessed in a repeated measures manner, the clinical application of ABA with functional analysis and discrete trial training in a naturalistic setting to increase the occurrence of mastered target behaviors and decrease problematic behaviors with a three-month snapshot (March 19, 2023, through June 11, 2023) sample [22]. Repeated measures deal with outcomes measured on the same experimental unit at different times or under other conditions, with each participant serving as their control [23,24]. 

Data analysis 

All descriptive and inferential statistics were performed using IBM SPSS Statistics for Windows, Version 29 (Released 2023; IBM Corp., Armonk, New York, United States) [25]. The significance level (α) was established at 0.05. The null hypothesis was rejected if the p-values were less than 0.05, indicating statistical significance. A summary of demographics and baseline characteristics were provided. Summary statistics were generated for the categorical variables, such as gender and race/ethnicity, and the continuous variables, including age, Time 1, Time 2, Time 3, Time 4, Time 5, Time 6, and Time 7 variables. These statistics for continuous variables included mean, standard deviation, median, and range. A series of two-independent sample t-tests were conducted to ascertain statistically significant mean differences between male and female groups at the two-week intervals across all seven timepoints. 

Inter-rater agreement 

A two-way random effects model was calculated, considering both the effects of individuals and measures as random. The intraclass correlation coefficient (ICC) two-way random effects model (2) was utilized, typically employed when multiple measurements are taken from each rater and then averaged. The ICC (2) value was found to be 0.860 (95% CI: 0.758-0.915), demonstrating excellent agreement among the raters. This value was higher than the average Pearson r (0.750), indicating that the ICC (2) was more sensitive to the variability among raters and measurements. The Cronbach’s alpha for the seven timepoint variables was r = 0.91, signifying a high level of internal consistency reliability [26,27]. 

IRB approval 

This study was carried out retrospectively using data gathered from clinical chart reviews. The research was reviewed and granted an exemption (#1-1703366-1) by the Western Copernicus Group-Institutional Review Board (WCG-IRB). The authors affirm that the analysis adhered to the ethical standards outlined in the 1964 Declaration of Helsinki and its subsequent amendments or equivalent ethical standards. It’s important to note that the Oxford Recovery Center (ORC), which obtained the ClinicalTrials.gov Identifier: NCT06043284, has since changed its name to The Oxford Center (TOC) (additional study ID numbers: OxRS-01-2021). 

## Results

Descriptive statistics

In a sample of 100 individuals with autism, the mean age was 8.88 years, with a standard deviation of 8.05 years and a median of seven. The youngest participant was one year old, and the oldest was 73. Data for seven participants was missing. Of the total, 74 (74.0%) were males and 25 (25.0%) were females, with one missing gender data (4:1). The sample contained 75 Whites (75.0%), four Asians (4.0%), three Hispanics (4.0%), 12 Middle Eastern (12.0%), and six African Americans (6.0%), with no missing ethnicity data. In terms of age groups, 18 (18.0%) were between one year old and four years old, 39 (39.0%) were between five and eight years old, 20 (20.0%) were between nine and 12 years old, 12 (12.0%) were between 13 and 16 years old, and four (4.0%) were between 17 and 73 years old. Four participants were over 17 years old, specifically 18, 20, 25, and 73 years. Data for seven participants was missing. 

Inferential statistics

Two independent sample t-tests were conducted to compare gender at seven time points at two-week intervals (Table 1).

**Table 1:** Two independent sample t-tests and effects sizes for cumulative target behaviors SD: Standard deviation; MD: mean difference; CI: confidence interval

Comparative analysis	Male mean (SD)	Female mean (SD)	t-value	Degrees of freedom	p-value	Mean difference (MD)	(MD)-CI (lower)	(MD)-CI (upper)	Effect size (d)	(d)-CI (lower)	(d)-CI (upper)
Time 1	1.060 (1.92)	2.050 (3.96)	-1.590	90	0.115	-0.989	-2.220	0.250	-0.390	-0.870	0.095
Time 2	3.710 (4.51)	4.070 (5.15)	-0.310	88	0.757	-0.355	-2.630	1.920	-0.076	-0.557	0.405
Time 3	7.095 (8.78)	8.610 (11.28)	-0.660	88	0.514	-1.518	-6.120	3.080	-0.160	-0.642	0.321
Time 4	13.170 (16.2)	13.070 (16.93)	0.026	88	0.980	0.105	-7.880	8.090	0.006	-0.474	0.487
Time 5	17.210 (18.86)	17.430 (22.17)	-0.045	87	0.965	-0.219	-9.780	-9.540	-0.011	-0.500	0.478
Time 6	21.007 (21.33)	20.680 (26.12)	0.059	88	0.953	0.326	-10.68	11.330	0.014	-0.466	0.495
Time 7	26.120 (24.22)	29.640 (33.74)	-0.536	89	0.593	-3.517	-16.54	9.510	-0.131	-0.611	0.349

## Discussion

The objectives of this study were twofold: (1) to provide supporting evidence for the 4:1 male-to-female ratio that is commonly cited in scientific research and (2) to determine whether a significant gender difference or lack thereof exists by evaluating general aggregate target behaviors across seven distinct timepoints at two-week intervals as the result of ABA interventions. Despite slight mean gender differences (female means slightly larger), this study found no statistically significant differences (p > .05) between male and female autistic individuals (Table 1) on all seven cumulative target behavior timepoint variables. These results suggest that the ABA therapies administered may have an equal impact on the research subjects regardless of whether they are male or female. However, it should be vehemently noted that the overall trend with wide CIs reported in this study and the statistical uncertainty associated with them may suggest substantial possible gender differences. CIs are values likely to contain the true population parameter within a specific range. When these intervals are wide, it indicates higher uncertainty or variability in the data. This statistical uncertainty, in turn, could hint at potential gender differences. The data might suggest that the study's outcome differs for all genders. There could be distinct patterns or trends for different genders, which is reflected in the variability captured by the wide CIs.

In terms of general gender disparities in ASD symptoms, Rivet and Matson found no significant gender differences in ASD symptoms in their infant/toddler or child/adolescent populations [28]. In the adult population, females had higher endorsements of social (i.e., participation in social games, sports, and activities; interest in other’s side of the conversation; and imitation) and communication (i.e., interest in other’s side of the conversation and reading body language) impairments compared to males [28]. Our study's female means were slightly higher throughout the seven timepoints but were not statistically significant. We also confirmed a 4:1 male-to-female ratio, as Loomes et al. reported, within our sample of research subjects [29].

Blair et al. suggested that the lack of gender difference findings in ABA’s impact on autistic individuals could be due to several factors [30]. Historically, autism research has been male-focused due to the higher autism prevalence in males. This bias has led to females and nonbinary individuals often being overlooked in studies, including those on ABA effects. Consequently, ABA’s impacts may not have been as thoroughly researched in females. Females with autism frequently receive misdiagnoses or additional diagnoses like bipolar disorder, depression, and anxiety, complicating the assessment of ABA effects and making it difficult to identify gender differences. The underrepresentation of females in research further hampers detecting and analyzing gender differences in ABA effects [30]. 

Moreover, neurodiversity emphasizes that every individual, including those on the autism spectrum, is unique. Therefore, the impacts of ABA might vary greatly among individuals, regardless of gender. Autistic individuals have unparalleled expertise in their own lives and their communities. Thus, the effectiveness of ABA could be influenced more by individual differences rather than gender differences [31,32]. Autism is a spectrum disorder with a wide range of symptoms and severity that can vary greatly among individuals. These individual differences can overshadow gender differences, making them difficult to detect and analyze in studies on the effects of ABA treatments [7]. Despite the lack of gender differences in our research, it is essential to note that individual responses to ABA treatments can vary widely. Factors such as age, cognitive level, and severity of autism symptoms may influence treatment outcomes. Future research should continue to explore these factors in more detail. 

As mentioned, research has consistently reported a higher rate of ASD diagnoses in males than in females. This could potentially lead to a bias in the research data, with more males being represented in ABA studies than females [29,31]. Some studies have found that female individuals with ASD had significantly better social interaction and social communication skills compared to male individuals with ASD. These inherent differences might influence the outcomes of ABA, making it difficult to directly compare the impacts of ABA between genders. There have been criticisms of ABA from the autistic community, which have had an appreciable effect on research, practice, and conversation in stakeholder groups. These criticisms often relate to the bias reflected within current practices, which have impeded the dignity and autonomy of many individuals with disabilities served through ABA. This could potentially limit the research on the impacts of ABA on different genders [31,32].

In addition, it is essential to note that while these factors might limit the availability of results on gender differences in the impacts of ABA, they also highlight the need for more inclusive and individualized approaches in ABA and autism research. Thus, this paper explored new ground. This study found no statistically significant differences (p > .05) between male and female autistic individuals on all seven cumulative target behavior timepoint variables with wide CIs which indicate a degree of uncertainty. Our results suggest that, statistically, ABA treatments, over time, may or may not affect both males and females similarly, which is consistent with the principles of ABA that emphasize individualized and function-based strategies. These findings underscore the importance of focusing on each individual's needs and characteristics rather than making gender-based assumptions. 

There is a growing recognition of the need for more inclusive research practices that take into account gender differences in autism. This includes the need for studies specifically delineating gender differences in the effects of ABA treatments. The lack of studies delineating gender differences in the impact of ABA treatments on autistic individuals is likely due to a combination of historical bias, diagnostic complexities, underrepresentation of females in research, individual differences in autism, and the need for more inclusive research practices. More research is needed to address these issues and improve our understanding of gender differences in the effects of ABA treatments on autistic individuals. This study contributes to the limited research on gender differences in ABA treatment outcomes by showing that ABA treatments may be equally effective for both genders with a degree of uncertainty. This study may provide valuable information for clinicians, educators, and parents. This research advocates for the application of ABA treatments to all individuals with autism, irrespective of their gender.

Limitations

This study has certain limitations. Our findings should be interpreted cautiously due to the limited number of studies in this area as well as several limitations in research reporting nonstatistically significant gender differences in autistic individuals. The large standard deviations computed resulted in wide CIs, which indicate high variability or uncertainty in our data with respect to a "true mean difference." The use of a nonrandom sample may limit this study's scope, and findings may not accurately reflect the entire population of individuals with ASD or a broader context. There is no ability to generalize beyond this sample. Additionally, identifying differences between groups and fully accounting for potential confounding factors presents a challenge. Another limitation is that this study’s outcomes can be influenced by several factors, including the variability in task stimuli, the number of trials, the participant types, the administration conditions, and the focal task variable. These elements add complexity and make it difficult to compare results across different studies.

Also, autism research was initially described and diagnosed primarily in boys and men, which may have led to an underrepresentation of females (smaller female sample sizes), resulting in a lower statistical power scenario relative to females. Research on gender differences, such as the present study, have focused on broad ranging variables, which may not capture subtle discrepancies inherent with the wide variation in ASD symptoms. Misdiagnosis or further diagnosis may occur in autistic individuals with co-occurring conditions such as anxiety, depression, and bipolar disorder. These may muddle the accuracy of measurements of the effects of treatment, making it difficult to ascertain gender differences.

These limitations highlight the need for more comprehensive and inclusive research practices in autism research. It’s important to consider these factors when interpreting the results of studies reporting nonstatistically significant gender differences in autistic individuals. While this study offers important insights, future research could improve upon these limitations by using a more diverse and randomized sample, maintaining consistent administration conditions, and continued validation of the instruments used. This approach would strengthen the study’s robustness and enhance the applicability of the results.

Further research is needed to confirm our results and to explore potential gender differences in other aspects of ABA treatment, such as parental involvement and treatment intensity. 

## Conclusions

This study’s findings on nonsignificant gender differences in response to ABA treatments may be noteworthy, suggesting that ABA treatments may be equally effective for both boys and girls with autism. Nevertheless, these results should be interpreted with caution. It is crucial to emphasize that the general pattern observed in this study, characterized by broad CIs, carries a degree of statistical uncertainty pointing toward substantial possible gender differences. Further research would be needed to confirm this hypothesis with an extension study and understand the nature of these potential differences. This study's findings may challenge pre-existing assumptions about gender differences in treatment response. This could have significant implications for clinical practice, suggesting clinicians should not preferentially recommend ABA treatments for one gender. Instead, treatment recommendations should be based on the individual needs and characteristics of each child, regardless of their gender. Our researchers hope that these findings will encourage further research in this area. Indeed, understanding the factors that influence treatment response is crucial for improving treatment outcomes and personalizing care. Future research could explore other potential moderators of treatment response, such as age, severity of autism symptoms, or co-occurring mental health conditions. This could ultimately lead to more personalized and effective care for children with autism. 
